# Mapping and quantifying unique branching structures in lentil (*Lens culinaris* Medik.)

**DOI:** 10.1186/s13007-024-01223-1

**Published:** 2024-06-19

**Authors:** Adam M. Dimech, Sukhjiwan Kaur, Edmond J. Breen

**Affiliations:** 1 Agriculture Victoria Research, Department of Energy, Environment and Climate Action, AgriBio Centre for AgriBioscience, Bundoora, VIC 3083 Australia; 2 School of Applied Systems Biology, La Trobe University, Bundoora, VIC 3083 Australia

**Keywords:** Lentil, Morphology, Structure, Phenotyping, Image analysis, Python, LemnaTec, PlantCV

## Abstract

**Background:**

Lentil (*Lens culinaris* Medik.) is a globally-significant agricultural crop used to feed millions of people. Lentils have been cultivated in the Australian states of Victoria and South Australia for several decades, but efforts are now being made to expand their cultivation into Western Australia and New South Wales. Plant architecture plays a pivotal role in adaptation, leading to improved and stable yields especially in new expansion regions. Image-based high-throughput phenomics technologies provide opportunities for an improved understanding of plant development, architecture, and trait genetics. This paper describes a novel method for mapping and quantifying individual branch structures on immature glasshouse-grown lentil plants grown using a LemnaTec Scanalyser 3D high-throughput phenomics platform, which collected side-view RGB images at regular intervals under controlled photographic conditions throughout the experiment. A queue and distance-based algorithm that analysed morphological skeletons generated from images of lentil plants was developed in Python. This code was incorporated into an image analysis pipeline using open-source software (PlantCV) to measure the number, angle, and length of individual branches on lentil plants.

**Results:**

Branching structures could be accurately identified and quantified in immature plants, which is sufficient for calculating early vigour traits, however the accuracy declined as the plants matured. Absolute accuracy for branch counts was 77.9% for plants at 22 days after sowing (DAS), 57.9% at 29 DAS and 51.9% at 36 DAS. Allowing for an error of ± 1 branch, the associated accuracies for the same time periods were 97.6%, 90.8% and 79.2% respectively. Occlusion in more mature plants made the mapping of branches less accurate, but the information collected could still be useful for trait estimation. For branch length calculations, the amount of variance explained by linear mixed-effects models was 82% for geodesic length and 87% for Euclidean branch lengths. Within these models, both the mean geodesic and Euclidean distance measurements of branches were found to be significantly affected by genotype, DAS and their interaction. Two informative metrices were derived from the calculations of branch angle; ‘splay’ is a measure of how far a branch angle deviates from being fully upright whilst ‘angle-difference’ is the difference between the smallest and largest recorded branch angle on each plant. The amount of variance explained by linear mixed-effects models was 38% for splay and 50% for angle difference. These lower R^2^ values are likely due to the inherent difficulties in measuring these parameters, nevertheless both splay and angle difference were found to be significantly affected by cultivar, DAS and their interaction. When 276 diverse lentil genotypes with varying degrees of salt tolerance were grown in a glasshouse-based experiment where a portion were subjected to a salt treatment, the branching algorithm was able to distinguish between salt-treated and untreated lentil lines based on differences in branch counts. Likewise, the mean geodesic and Euclidean distance measurements of branches were both found to be significantly affected by cultivar, DAS and salt treatment. The amount of variance explained by the linear mixed-effects models was 57.8% for geodesic branch length and 46.5% for Euclidean branch length.

**Conclusion:**

The methodology enabled the accurate quantification of the number, angle, and length of individual branches on glasshouse-grown lentil plants. This methodology could be applied to other dicotyledonous species.

**Supplementary Information:**

The online version contains supplementary material available at 10.1186/s13007-024-01223-1.

## Background

Lentils (*Lens culinaris* Medik.), a member of the Fabaceae legume family, is an annual grain legume crop cultivated globally for its high dietary benefits. Rapidly increasing populations in developing countries have led to an increased demand for pulses, including lentils, as a protein source in diets. Furthermore, there has been a noticeable shift in dietary habits in Western countries, with a growing preference for nutritious, sustainable, and healthier foods. Lentils, with their rich nutrient profile and eco-friendly cultivation practices, align perfectly with these changing consumer preferences. According to [1], global lentil production in 2022 reached 6.8 million tonnes from an estimated 5.5 million hectares, with an average yield of 1218 kg ha^−1^. Canada is the major producer of lentils, followed by India, Australia, and Turkey. However, Australia takes the lead in the export market, with over 95% of its total lentil production being exported. The Australian lentil industry emerged in the 1990s, with South Australia and Victoria serving as major production zones. Over the years, the demand and export value of Australian lentils have increased significantly. Australian lentil production scaled up to 1.68 million tonnes in 2022, compared to a mere 0.03 million tonnes produced in 1990 [2].

The widespread and increased cultivation of lentils in Australia has been facilitated by extensive plant breeding efforts over successive decades, which have significantly contributed to yield gains [3]. Over time, breeders have focused on improving disease resistance, abiotic stress tolerance, plant phenology and seed quality traits, all leading to better and stable yields. Recent studies suggest that crop architecture has also played a key role in yield gains in Australian national lentil breeding programs over the past 27 years and increased plant height and leaf size along with reduced branching, changes that are positively correlated with yield. Whilst breeders have been modifying crop architecture through the direct selection of these traits, they were not correlated with the year of variety release, which indicates the usefulness of these traits for increasing yield has not yet been fully realised in Australian lentil breeding programmes [3]. Accurate selection in plant breeding programs relies heavily on precise phenotyping, which involves the quantitative measurement of various complex traits such as growth, development, tolerance, resistance, architecture, physiology, and yield [4]. In recent years, there have been significant advancements in image-based plant phenomics, enabling researchers to non-destructively phenotype traits across a wide range of plant species. This has been made possible through the utilisation of high-throughput automated phenotyping platforms like the LemnaTec Scanalyser 3D. The development of image-based plant phenomics has revolutionised the field, offering researchers the ability to accurately measure biomass accumulation, growth patterns, changes in nutrient status, and stress responses in crop plants. One of the major advantages of these automated platforms is their ability to collect time-course data in a consistent and quantitative manner. The introduction of hyperspectral [5] and fluorescence [6] imaging technologies has significantly expanded the range of traits that can be measured in plant phenotyping, allowing researchers to capture and analyse a broader spectrum of information related to plant physiology, biochemical composition, and stress indicators. Despite the increasing popularity of these advanced imaging modalities, red–green–blue (RBG, i.e.: visible-spectrum) imagery remains the most prevalent and practical choice in plant phenotyping systems.

Until now, none of the technologies and associated software have been able to reliably measure architectural traits such as branching, a valuable morphological trait in complex dicotyledons. In contrast, there has been some progress in monocotyledonous species such as barley [7], wheat [8] and corn [9], to quantifying leaf architectural traits such as angle, quantity, and length. Analysing the structure of thalli from the liverwort *Riccardia longispica* is another example of using morphometrics to describe plant structure [10]. Recent examples of phenotyping dicotyledonous species have continued to rely on manual counts [11] rather than a programmatic approach as described here.

This paper describes a methodology to quantify the number of branches in lentil plants via RGB imagery and derive some simple statistics including length (both Euclidean and geodesic), and branch angle. The experiments described here aimed to (i) develop an algorithm for extracting branching data from RGB images of lentils; (ii) test the algorithm on diverse lentil lines to demonstrate reliability and accuracy; and finally (iii) test whether the algorithm could be used to discriminate between different lentil lines based on their growth habit or exposure to salt as a demonstration of a practical application.

## Methods

### Glasshouse conditions

**Fig. 1:**
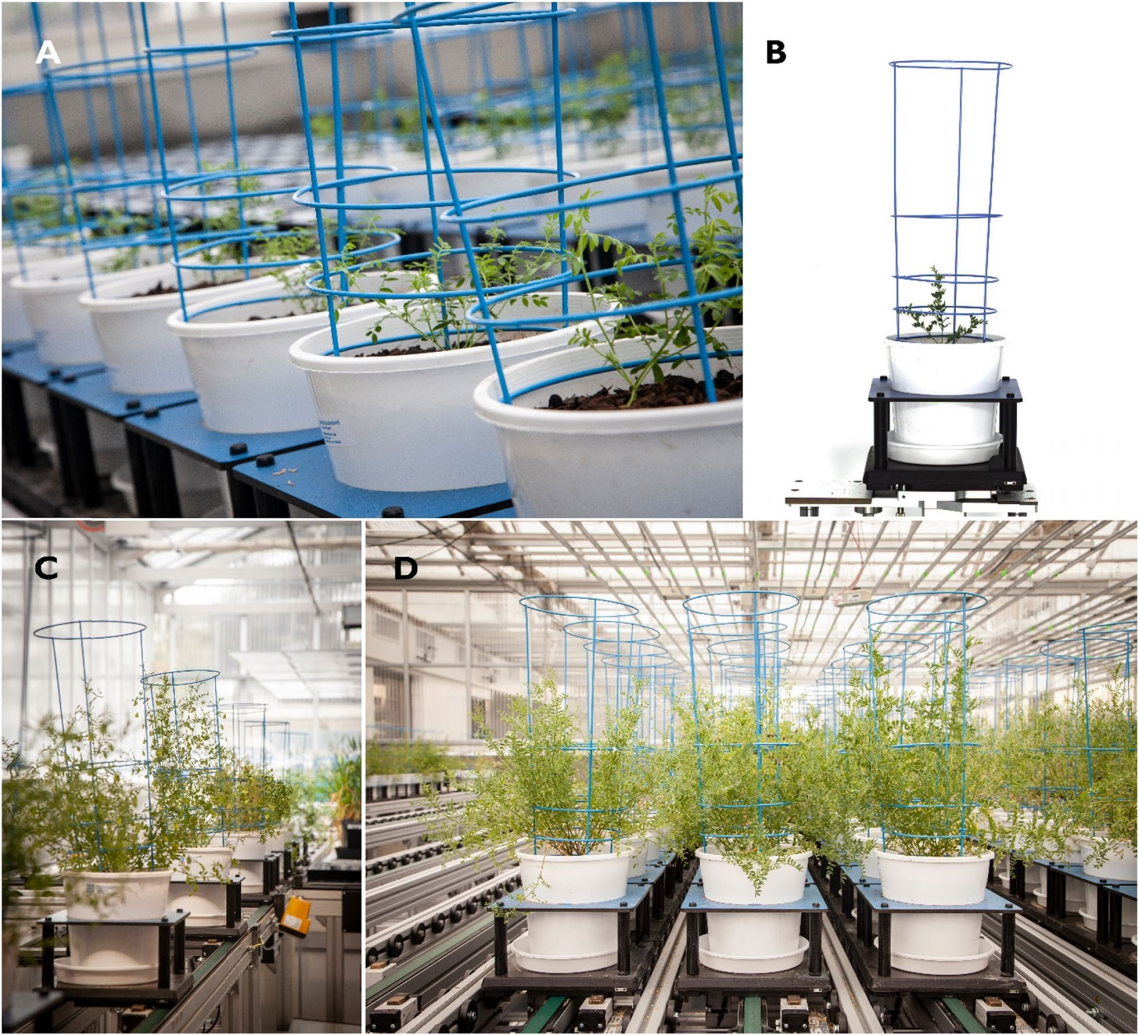
Lentils growing at “Plant Phenomics Victoria”: **A** Young lentil plants growing in white 200 mm diameter pots with blue support cages on the LemnaTec Scanalyser 3D phenomics platform; **B** a typical RGB image of a lentil plant captured by cameras in the LemnaTec Scanalyser 3D phenomics platform; **C** lentil plants being moved from the glasshouse to the imaging cabinet via conveyors; **D** mature lentil plants on the LemnaTec Scanalyser 3D phenomics platform at the conclusion of Experiment 1

Two glasshouse experiments were conducted at ‘Plant Phenomics Victoria’ in Bundoora, Victoria, Australia (− 37.724251, 145.056586). Experiment 1 was conducted for two months from August 2020 (Fig. 1); Experiment 2, which comprised a re-analysis of data from an earlier experiment, was conducted for 3 months from July 2019.

Seeds from diverse cultivars and lines (hereafter referred-to collectively as genotypes to prevent confusion with ‘lines’ in image analysis) from the Australian Lentil Breeding Program were germinated in white 200 mm diameter plastic pots (catalogue P200E04, Garden City Planters Pty. Ltd., Dandenong South, Victoria, Australia) using a commercial potting mix blend that contained coir peat, composted pine bark, composted sawdust, SaturAid® soil-wetting agent, lime and gypsum (Australian Growing Solutions Pty. Ltd., Tyabb, Victoria, Australia). The potting mix was supplemented with iron chelate and Green Jacket® 9-month controlled-release fertiliser (18:2.5:10, Australian Growing Solutions Pty. Ltd., Tyabb, Victoria, Australia). Three seeds were sown to a depth of 50 mm per pot, then thinned at 5 days to leave a single plant per pot. To provide support for emerged seedlings, a wire ‘cage’ powder-coated with a blue (Reichs-Ausschuss für Lieferbedingungen [RAL] 5012 ‘Light Blue’) resin was placed in each pot at the time of sowing. Watering was conducted daily and targeted to 80% soil gravimetric water content.

The pots were each placed in a carrier which contained a radio frequency identification (RFID) chip and loaded onto a LemnaTec Scanalyzer 3D (LemnaTec GmbH, Aachen, Germany) high-throughput phenomics platform. Seedlings were grown in a climate-controlled glasshouse at 22 °C from 7:00 to 20:00 and 15 °C from 20:00 to 7:00. Supplemental light was provided by full-spectrum ‘white’ overhead light-emitting diodes (Photon Systems Instruments spol. s r.o., Drásov, Czech Republic) during daylight hours that provided a photosynthetic photon flux of 600 µmol m^−2^ s^−1^. The glasshouse was clad in ‘plexiglass’ (poly(methyl methacrylate)) double-walled sheeting that allowed the full spectrum of sunlight to pass through.

#### Experiment 1

A random block design was used, where each block consisted of 19 positions with a single replicate of each line. Each physical row on the LemnaTec Scanalyser 3D platform contained two blocks, and these extended over 5 lanes to contain 10 blocks of 190 pots in total. A random number generator in R (version 4.3.1), was used to assign each genotype to a position in the block.

Nineteen genotypes were selected for detailed imaging based on their diverse characteristics; ‘Aldinga’, ‘CDC Ruby’, ‘CIPAL0717’, ‘Cobber’, ‘Commondo’, ‘Cumra’, ‘Digger’, ‘Eston’, ‘ILL2024’, ‘ILL7537’, ‘Indianhead’, ‘Matilda’, ‘Nipper’, ‘Northfield’, ‘PBA Bolt’, ‘SP1333’, ‘PBA Hallmark XT’, ‘PBA Jumbo2’ and ‘PBA Greenfield’ (refer to SI01).

#### Experiment 2

The experimental methodology for Experiment 2 is described as “Experiment 3” in [12]. In summary, 276 lentil accessions were grown under identical conditions to Experiment 1 but exposed to one of two salt conditions: control (0 mmol NaCl) and salt (100 mmol NaCl). A partial replication design was used to screen 912 plants. A complete list of the genotypes used in Experiment 2 are detailed in SI02. Images from Experiment 2 were re-analysed in PlantCV to demonstrate a practical application for the current work by determining whether salinity would affect branch architecture data and whether this could be quantified using the approach described.

### Collection of images

For Experiment 1, plants were imaged daily on the LemnaTec Scanalyser 3D automated phenomics platform via a series of visible-spectrum (red–green–blue, RGB) cameras (Prosilica GT, Allied Vision Technologies GmbH, Stadtroda, Germany) fitted with a 50 mm focal lens (T* 250 ZF, Carl Zeiss AG, Oberkochen, Germany), located within an imaging cabinet to control lighting conditions. RGB images of each plant were taken from above (top view, TV) and from the side at five angles (0°, 20°, 40°, 60°, 80°) as per the methodology of [2]. Imaging for Experiment 2 was collected several times a week and from only two angles (0°, 90°) as described in [12].

Snapshot images were stored in a PostgreSQL database as blob files. A modified version of PlantCV ‘Data Science Tools’ was used to extract each snapshot from the database as a series of six 24-bit Portable Network Graphic (PNG) images measuring 4384 × 6576 pixels.

### Definition of a branch

A lentil “branch” was defined as an outgrowth from the primary stem or the base of the plant marked by an internode extension reaching about 80% of the length of other adjacent internodes and at least half of the pinnae had opened on the first leaf. Any branches that were visible by manual inspection but located below the upper rim of the pot were excluded from the count.

### Image processing and analysis

A customised PlantCV [13] analysis pipeline was written to perform the image analysis steps. PlantCV version 3.12.0 with OpenCV version 3.4.10.35 and Python version 3.8.2 under CentOS Linux version 7.9.2009 operating system was used on the Biosciences Advanced Scientific Computer (BASC) at the Centre for AgriBioscience in Bundoora, Victoria, Australia.

**Fig. 2:**
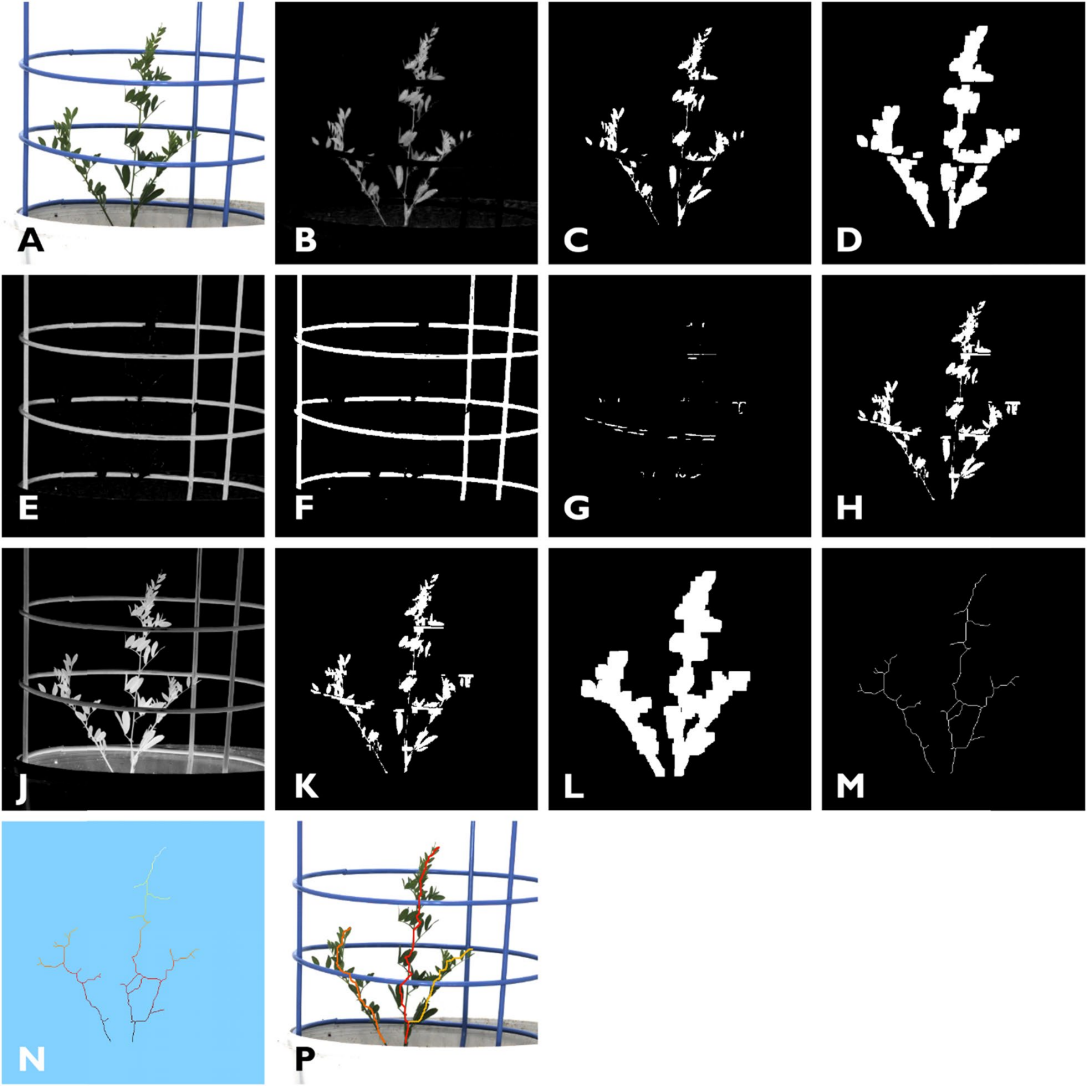
Representation of stages in the image analysis pipeline to identify unique branches in lentils: **A** a cropped section of the original RGB image; **B** the ‘Y’ channel from a CMYK transformation of the original RGB image, this gives a greyscale representation that is used to help isolate the plant from its background. **C** A binary image derived from the Y channel, thresholded at grey level 63. **D** A 9 × 9 pixel dilation applied to the binary image to bind some of the objects; **E** the ‘M’ channel from CMYK transformation of the original RGB image, this gives a greyscale representation which is used to isolate the cage; **F** a binary image derived from the M channel, thresholded at grey level 55; **G** a pixel-wise logical-AND operation applied between the thresholded images of the plant and the cage to identify pixels that overlap the cage and the plant so as to restore the parts of the plant occluded by the cage; **H** binary image of the plant without the cage; **J** the ‘K’ channel from a CMYK transformation of the original RGB image that gives a greyscale representation and is used to help isolate any visible potting mix. This is thresholded to create a binary image [not shown]; **K** the potting mix is subtracted from the plant mask via the application of a pixel-wise logical-AND operation between an inverted potting mix mask and the plant mask; **L** a 13 × 13 pixel dilation applied to the binary mask; (M) A skeleton derived from the binary shape in **L**. Note the two disconnected components; **N** a heatmap showing the distance of each pixel from the top of the pot where greater distance values map to lighter pixel values; **P** the original RGB image superimposed with unique branch lines derived from the skeleton when all loops are removed, lateral branches clipped and small independent branches restored

PNG images from each snapshot were read into the program and processed (Fig. 2). Images were rotated 90° and cropped to standardise them for further processing. The RGB images (Fig. 2A) were converted to CMYK colour space (cyan, magenta, yellow, and black bands) [14]. The Y channel (Fig. 2B) was found to be useful for separating the plant from its background, and as the lighting was held constant across all images, this was easily accomplished by thresholding it at a constant grey-level (Fig. 2C). Next, a 9 × 9 dilation was applied to this binarisation to help fuse isolated plant sections (Fig. 2D). Similarly, by applying a threshold to the M channel (Fig. 2E) the blue plant support cage could be isolated (Fig. 2F). To further extend the fusing of isolated plant sections; first, a pixel-wise logical-AND operation was applied between (Fig. 2C and F) producing Fig. 2G; and second on Fig. 2G, the following cascade was applied: a 9 × 9 erosion followed by an 11 × 11 dilation, followed by a pixel-wise logical-AND with Fig. 2C producing the result shown in Fig. 2H.

**Fig. 3:**
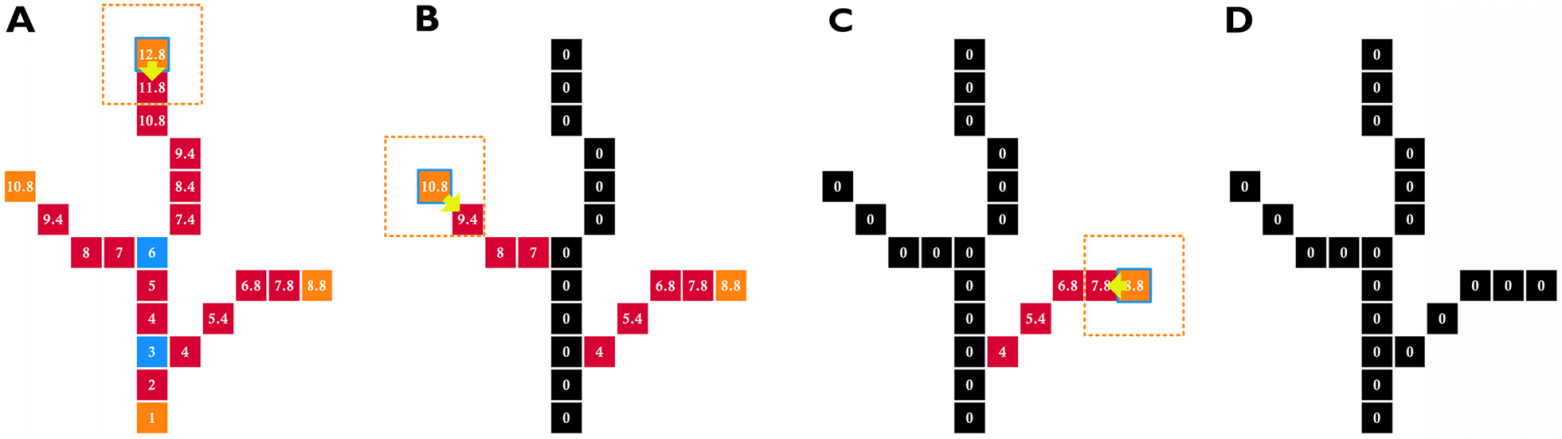
Schematic representation of a simplified binary skeleton that is being processed to identify unique branches which are branches that reach the base (nominally the level of the potting mix). **A** A skeleton consisting of 23 pixels comprising of one main branch with one left and one right lateral branches. Note that the base of the skeleton is highlighted with a geodesic distance value of 1. Endpoint pixels are displayed in orange, slab pixels are displayed in pink/red and junction pixels are displayed in blue. Each pixel has a geodesic distance value measured from the base point (shown as a number within each pixel). A queue-based algorithm processes each branch sequentially, starting at the endpoint of the longest branch (highlighted with a yellow arrow). The tracking process, which has a current position indicated by a pixel with a blue border, sets each visited pixel to 0 and then progresses down the branch using the 8-connected neighbourhood (dotted orange box) to identify the neighbour with the minimum distance value before moving to that position. **B** All of the pixels in the main branch have been set to zero. Note that the tracking process terminated at a pixel with the geodesic distance value of 1; therefore, it is a unique branch. The starting condition of the left lateral branch is highlighted. **C** All of the pixels in the left branch have been set to zero and the tracking process terminates at a pixel with a distance value of 7, which is greater than 1. Note that the starting condition of the right lateral branch is highlighted. **D** All pixels in the right branch have been set to zero. Note that the right branch terminates at a pixel with a distance value of 4, which is greater than 1

The algorithm automatically separated any potting mix from the image in a pre-processing step; the K channel (Fig. 2J) from the CMYK transformation was inverted, and a static threshold was applied (Fig. 2K). A logical-OR operation was then used to create a new mask that excluded the potting mix, focusing solely on the plant. A 13 × 13 dilation was applied to achieve the final mask size (Fig. 2L). Subsequently, a skeletonisation [15] was applied (Fig. 2M) to facilitate the identification of the plant’s branch structures. As shown in Fig. 2M, it is possible for a plant’s skeleton to be composed of disconnected segments, therefore a connected component analysis was used to label each skeletal component. The skeleton image is composed of pixels classified into two groups (0 and 1, OFF and ON). The pixels composing the skeleton can be further classified into at least three categories (endpoints, junctions, and slabs). Endpoint pixels have only one ON neighbour; slab pixels have only two ON neighbours and junction pixels have more than 2 ON neighbours (Fig. 3A).

The lowest point on each skeleton was identified as the pixel with the greatest *y*-value, as images have coordinate (0,0) at the top left corner with *y* increasing top-to-bottom and *x* increasing left-to-right. The (*x*,*y*) position of the lowest point was added to a vector called *base*. When there were multiple pixels with an equally maximal value of *y*, such as when a skeleton had multiple components (for example, Fig. 2M), multiple points were added to the vector (*base*).

**Fig. 4:**
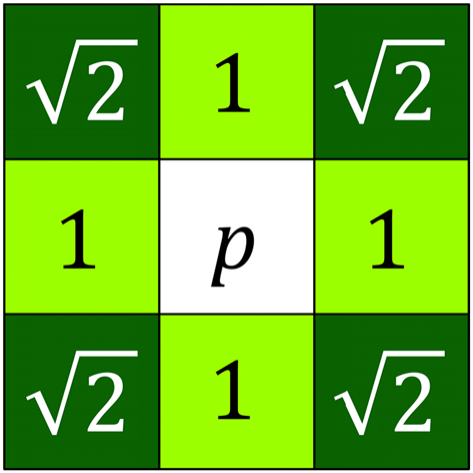
Representation of a pixel neighbourhood around a pixel at position *p*. 4-connected pixels have a distance value of 1 (light green) whilst 8-connected pixels have a distance value of √2 (dark green) in accordance with Euclidean geometry

A geodesic distance is a distance measure that is constrained to lie within a certain path, in this case within the skeleton [16]. To determine geodesic distance values, an 8-connected neighbourhood array was defined. Within the neighbourhood of each pixel, 4-connected neighbours had a distance of 1, whilst the 8-connected neighbours had a distance of $\sqrt 2$ (Fig. 4) from the centre pixel. A First-In-First-Out (FIFO) queue-based algorithm and a neighbourhood function were used to assign geodesic distance values to pixel positions within the skeleton from the base point of each skeleton component (refer to Algorithm 1). These geodesic distance values were collected within a 32-bit single-precision floating-point format (float32) Portable Network Graphic (PNG) image of the same dimensions (Algorithm 1, Line 7).

**Algorithm 1:**
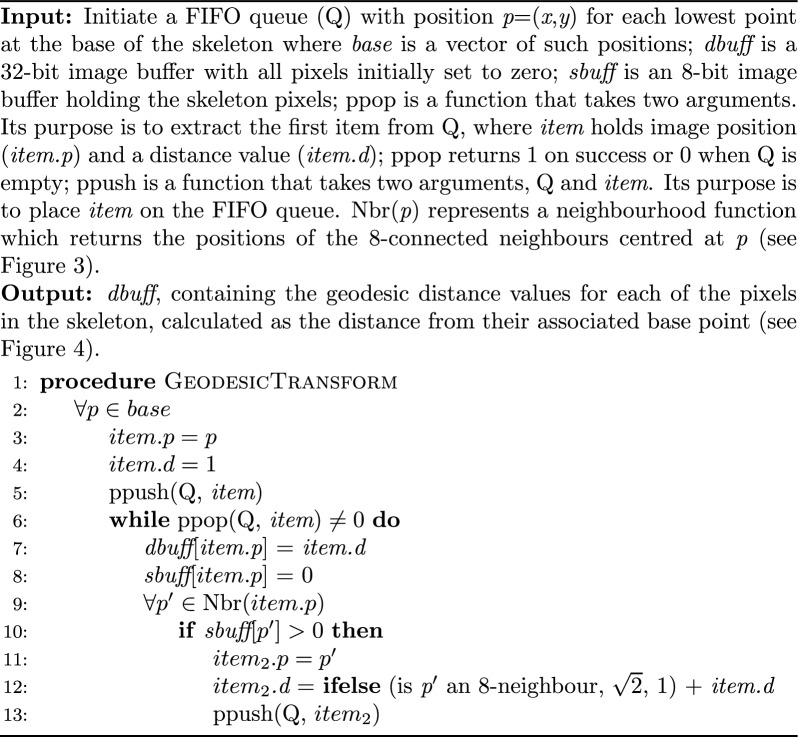
Queue based geodesic distance transform

The geodesic transformation, outlined in Algorithm 1, begins at the base pixel of the skeleton (Line 2). It then pushes this pixel with a starting distance of 1, on to the queue (Line 5). The algorithm then iteratively pops items off the queue (Line 6) and pushes their neighbouring pixels onto the queue (Line 13) until all skeleton pixels have been processed. To prevent backward iterations, the algorithm sets visited pixels to zero (Line 8). Neighbouring pixels that have a value of 1 (non-background pixels) are checked and added to the queue (i.e., non-background pixels; Lines 9–10). The collected pixel positions and distance values (*x*, *y* and *distance*) are added to the queue with an updated distance value 1+*item.d* for 4-connected neighbours, $\sqrt 2$+*item.d* for 8-connected neighbours as shown in Algorithm 1, Line 12. These collected distance values can be used to generate a heatmap graphic for inspection, as shown in Fig. 2N.

To collect the plant branches, the skeleton endpoints (*x*, *y*) that had a geodesic distance greater than 1 (Fig. 3A) were collected and stored in a vector called dval_skel. Then Algorithm 2 was used to iterate over these skeleton endpoints (Line 2) to identify two types of branches: (1) the longest unique branches (which are branches within each skeleton that extended from a skeleton endpoint to the base of the plant) and (2) significant lateral branches (which are branches that are at least 40% of the length of the longest branch, see Line 18). Note, as the image scale is held constant, distance values are comparable across images. Further, a test was applied to the skeleton endpoints in the list; if there was more than one skeleton, the minimum length required for a branch to be retained was set at 100 pixels, otherwise it was set to 40 to ensure the retention of small emergent seedlings whilst eliminating insignificant lateral branches.

The Algorithm 2 traces a pathway following the lowest pixel distance values to the base (Lines 7–20). To protect the algorithm from getting trapped in a loop, visited pixels are set to zero (Line 8). This procedure has the benefit of isolating lateral branches from the skeleton (Fig. 3) and implicitly removing loops (an example of a skeleton with two loops is shown in Fig. 2M and N).

**Algorithm 2:**
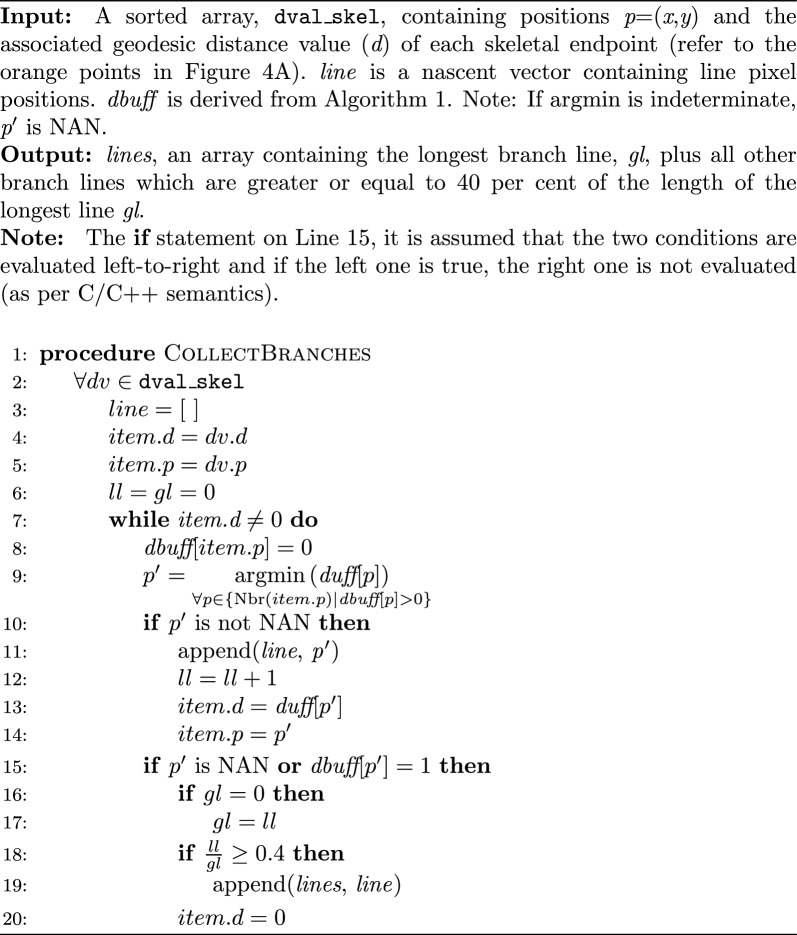
Collection of skeleton branches

### Data analysis and validation

For each identified branch its length was determined by a Euclidean distance between the start and endpoints of the branch, $d = \sqrt {\left( {x_{2} - x_{1} } \right)^{2} + \left( {y_{2} - y_{1} } \right)^{2} }$, where (*x*, *y*) represents image position. To calculate branch angle (*α*), angles were restricted to the range [0°,180°):

$$\begin{aligned} \theta & = \tan^{ - 1} \left( {\frac{{y_{2} - y_{1} }}{{x_{2} - x_{1} }}} \right) \\ \alpha & = \left\{ {\begin{array}{*{20}ll} {\theta + 180} & {{\text{if}}\;\theta < 0} \\ {\theta - 180} & {{\text{if}}\;\theta > 0} \\ \theta & {{\text{otherwise}}} \\ \end{array} } \right. \\ \end{aligned}$$

These two values (*d* and α) were added to the CSV file output along with the geodesic distance of the branch endpoint (Algorithm 2) and other branch metadata (such as a unique branch ID, plant ID and snapshot timestamp).

#### Validation of algorithm

Manual scoring was conducted on 463 images isolated from snapshots taken at 4 different developmental stages; 22, 29, 36 and 43 days after sowing (DAS) at both 0° and 90°, to confirm the reliability of the method and test whether the accuracy of the algorithm diminished with plant maturity. The images were examined to determine the branch count, after which the identical images were subjected to algorithmic analysis to compare results.

To verify the accuracy of the angles, approximately 10% of the branches were manually examined by overlaying a line at the respective angle onto each branch, ensuring alignment, and achieving a consensus of results.

#### Statistical analysis of Experiments 1 and 2

Statistical analyses were all performed using *R* v4.3.1. Mixed-effects models were used to model experimental responses. Analysis of Variance (ANOVA, from the car package) was used to test the significance of the models, where statistical significance was indicated at the 5% level.

While a Poisson model is normally used to describe the probabilities for count data, we observed a significant difference between the mean of the count distribution to that of a Poisson distribution with the same mean (the χ^2^ *p*-value was 0.0005). Therefore, to model the branch counts in Experiment 1, the following R model was used:

1 $${\tt{branch}}\_{\tt {count}}\sim {\tt {genotype}} + {\tt {DAS}} + {\tt{genotype:DAS}} + \left({\tt{1}|{\tt{plant}}\_{\tt{id}}} \right)$$

where genotype is a factor with 19 levels, DAS is a factor with 28 levels, and genotype:DAS is an interaction with 526 levels instead of 532, because 6 cultivars had no reading on Day 6 and plant_id is a factor with 154 levels. The expression (1|plant_id) specifies an independent random intercept to account for repeat readings of the plants in each genotype. The linear mixed-effects model in Eq. 1 was modified to predict the other response variables in Experiment 1.

To model the branch counts in Experiment 2, the following R model was used:

2 $${\tt{branch}}\_{\tt{count}}\sim {\tt{genotype}} + {\tt{DAS}} + {\tt{treatment}} + {\tt{genotype}}:{\tt{DAS}} + {\tt{genotype}}:{\tt{treatment}} + {\tt{DAS: treatment }} + \left({\tt{1}|{\tt{plant}}\_{\tt{id}}} \right)$$

where genotype is a factor with 276 levels, DAS is a factor with 5 levels, and treatment is a factor with 2 levels, genotype:DAS is an interaction with 1385 levels, genotype:treatment is an interaction with 554 levels, DAS:treatment is an interaction with 10 levels and plant_id is a factor with 908 levels. The expression (1|plant_id) specifies an independent random intercept to account for repeat readings of the plants in each genotype. The linear mixed-effects model in Eq. 2 was modified to predict the other response variables in Experiment 2.

#### Derivative factors for analysis

Two derived statistics were calculated: ‘splay’ and ‘angle-difference’.

Splay is a measure of how far a branch angle deviates from being fully upright. Since branch angles are recorded as values between 0° and 180°, which are relative to the image’s perspective, each recorded branch angle (α) was rescaled (α′) between 0° and 90°.

$$\alpha^{\prime} = \left| { - 90 + \frac{{\left( {\alpha - \min \left( \alpha \right)} \right)\left( { - 90 - 90} \right)}}{{\left( {\max \left( \alpha \right) - \min \left( \alpha \right)} \right)}}} \right|$$

Splay was then calculated as the maximum of α′ for each plant on any day.

Angle-difference is the difference between the smallest branch angle (min(α)) and largest branch angle (max(α)) where 0° ≤ α < 180° on the same day. This metric was designed to account for occasional lop-sidedness.

#### Salt tolerance classifications

Each lentil plant in Experiment 2 was given a score for salt tolerance (based on a physical inspection) as determined from a previous study [12]. Each plant was scored from 1 (healthy) to 10 (dead) [17]. In the present study, this data was reanalysed so that a median score for each cultivar was calculated and then classified as belonging to one of five broad salt-tolerance categories; (a) Highly Tolerant; (b) Tolerant; (c) Moderately Intolerant; (d) Intolerant or (e) Highly Intolerant.

## Results

### Experiment 1

#### Number of branches

The Lentil Branching algorithm exhibited promising performance in the detection of the number of branches in young plants grown on the LemnaTec Scanalyser 3D phenomics platform. However, as the plants reached maturity, the accuracy declined and the spread of errors, detected as an under- or over-estimation of branch counts when compared to a manual count, increased. Absolute accuracy, which allowed no deviation from the manual branch count, was 77.9% for plants at 22 days after sowing (DAS), 57.9% at 29 DAS and 51.9% at 36 DAS. With a deviation of ± 1 branch from the manual count, the associated accuracies were 97.6%, 90.8% and 79.2% respectively. Details of the spread of under- and overestimations in branch counts are detailed in Table 1.

**Table 1:** Discrepancies between manual branch counts and automated branch counts for lentil images collected in Experiment 1 at 22, 29, and 36 days after sowing (DAS) from a LemnaTec Scanalyser 3D phenomics platform

DAS	*n*	Number of discrepancies	Number of underestimations	Number of overestimations
22	154	34 (22.1%)	33 (21.4%)	1 (0.6%)
29	152	64 (42.1%)	62 (40.7%)	2 (1.3%)
36	154	74 (48.1%)	68 (42.1%)	6 (3.9%)

Counts are shown with percentages in parenthesis

**Fig. 5:**
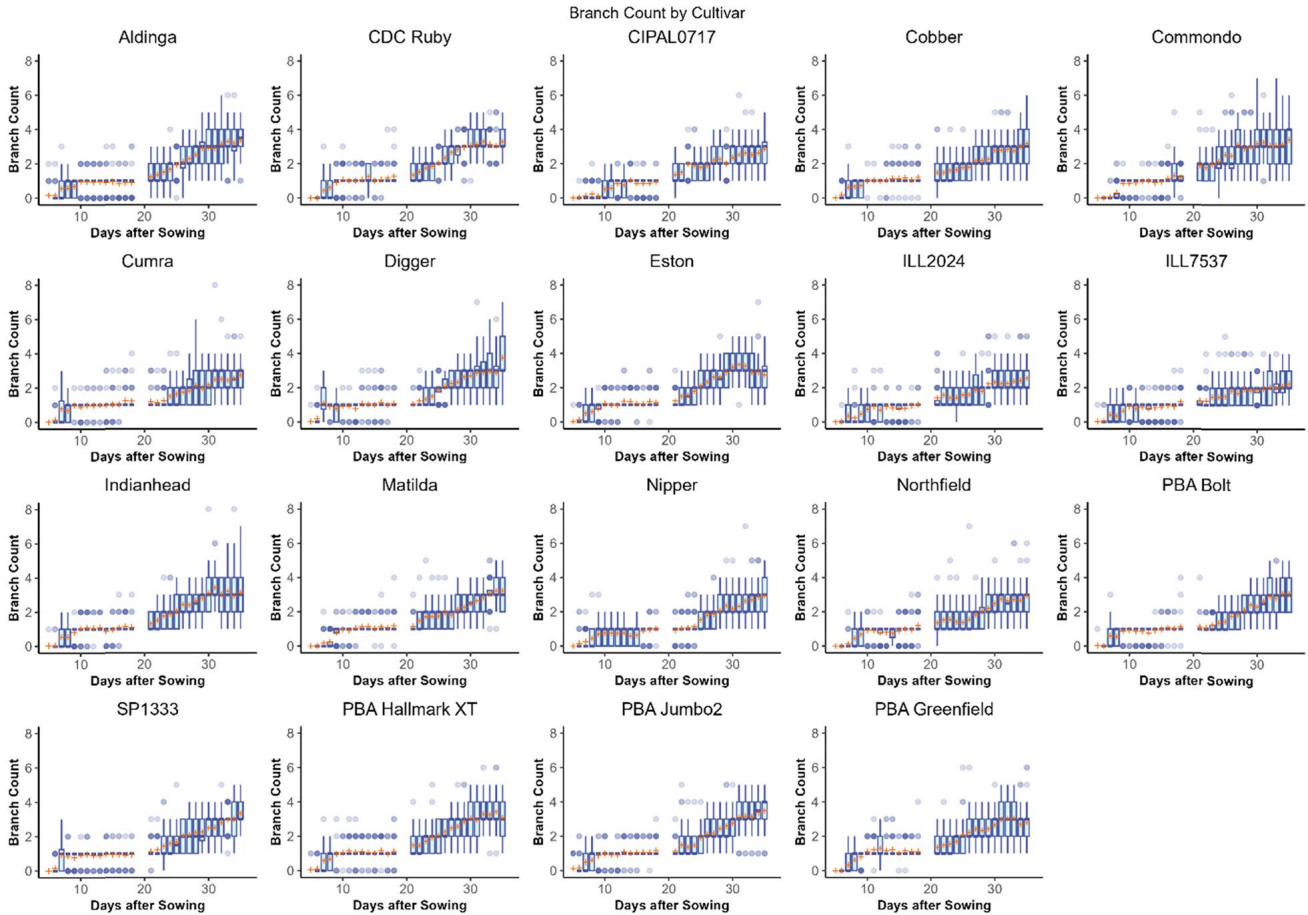
Box plots showing the distribution of mean number of branches counted by the lentil branching algorithm in Experiment 1 for each lentil cultivar at 0°, 20°, 40°, 60° and 80° imaging angles which have been pooled. The mean value is indicated with an orange cross. Across the experiment, the image angle at which an image was taken was not found to have a significant effect on branch counts (p = 0.5751)

**Fig. 6:**
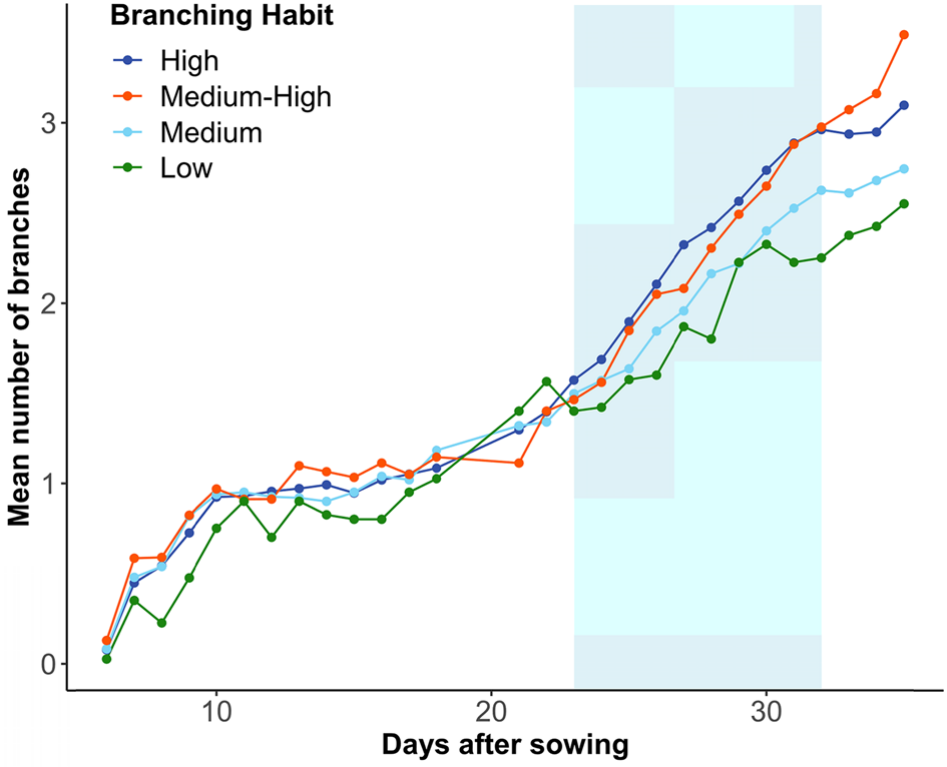
Observed mean number of branches per plant over time counted by the branching algorithm in Experiment 1 for lentil plants grouped into classifications based on previously-reported branching phenotypes as shown in the legend. Note that from days 23 to 32 (shaded), the reported classifications accorded with the measurements collected by the algorithm. The phenotype data was collected from field trials over several years conducted by Agriculture Victoria

Across the entire experiment, the mean number of branches measured for all genotypes increased between 5 to 35 DAS (Fig. 5). When the genotypes were classified into four branching habit classification groups based on their previously-documented phenotype for branching habit (refer to the “branching number” data in SI01, Fig. 6), DAS and branching habit classification were highly significant factors (p < 0.001). A pairwise t-test comparison of branching habit classifications found no significant difference between the Medium–High and High groups (p = 0.584, see blue and green lines in Fig. 6) but all other comparisons between the branching habit groups were significant.

As expected, genotype and DAS were found to have a significant effect on the mean number of branches (p < 0.01). After the experiment (35 DAS), cultivar ‘Digger’ had the greatest mean number of branches (3.75), followed by ‘PBA Jumbo2’ (3.488), ‘Commondo’ (3.40) and ‘Aldinger’ (3.376), however, there was no significant difference between the mean number of branches for ‘Digger’ as compared to the other aforementioned genotypes. In contrast, the genotype with the smallest mean branch count at 35 DAS was ‘ILL7537’ with a mean of 2.250 (Table 2).

**Table 2:** Summary of analysis of variance (ANOVA, Type II Wald chisquare test) results for Experiment 1

Response variable	R^2^	Factor	χ^2^	DF	Pr(> χ^2^)	*q*-value
Branch count	0.614	Cultivar	261.11	18	< 2.2e−16	3.3e−15
		DAS	29,914.92	1	< 2.2e−16	3.3e−15
		Cultivar:DAS	513.06	18	< 2.2e−16	3.3e−15
Geodesic length	0.824	Cultivar	79.784	18	9.35e−10	1.87e−09
		DAS	20,422.71	1	< 2.2e−16	3.3e−15
		Cultivar:DAS	1316.397	18	< 2.2e−16	3.3e−15
Euclidean length	0.868	Cultivar	67.935	18	1.01e−07	1.01e−07
		DAS	13,589.36	1	< 2.2e−16	3.3e−15
		Cultivar:DAS	1067.311	18	< 2.2e−16	3.3e−15
Angle difference	0.496	Cultivar	97.727	18	5.767e−13	2.3068e−12
		DAS	3104.39	1	< 2.2e−16	3.3e−15
		Cultivar:DAS	193.856	18	< 2.2e−16	3.3e−15
Splay	0.378	Cultivar	91.054	18	9.333e−12	2.7999e−11
		DAS	1664.174	1	< 2.2e−16	3.3e−15
		Cultivar:DAS	181.01	18	< 2.2e−16	3.3e−15

*q*-values are Holm [38], a family-wise error rate, multiple test-corrected *p*-value. Holm is generally considered to be a more powerful correction than the Bonferroni correction method

#### Branch lengths

**Fig. 7:**
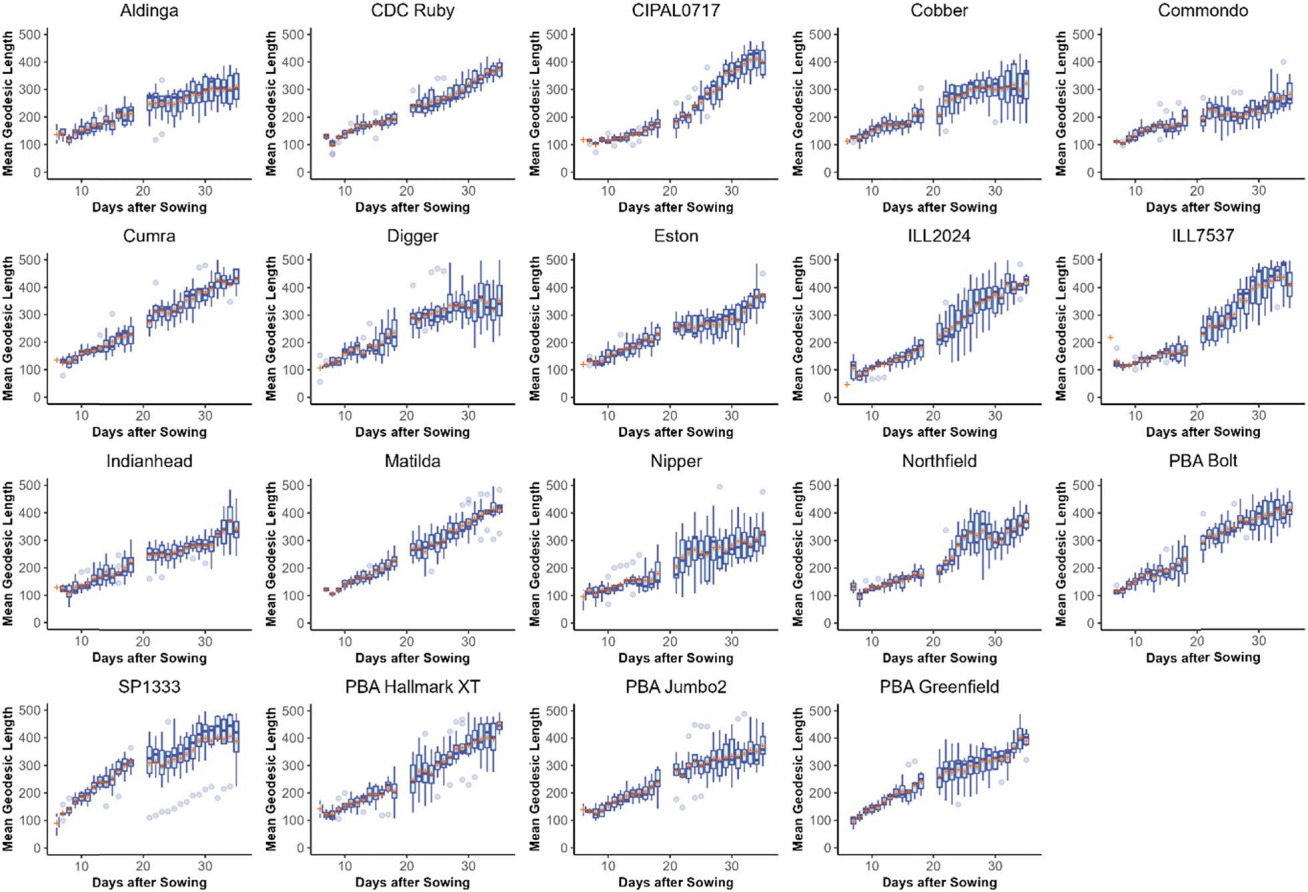
Box plots showing the distribution of geodesic lengths of branches counted by the branching algorithm in Experiment 1 for each lentil cultivar. The mean value is indicated with an orange cross. Note that the geodesic distances of branches increase over time for all cultivars and differ between cultivars. The break in the data on days 19 and 20 was due to a mechanical failure which prevented the collection of images on those days

The amount of variance explained by the linear mixed-effects models was 82% for geodesic branch lengths and 87% for Euclidean branch lengths. Within these models, both the mean geodesic (Fig. 7) and Euclidean distance (refer to SI03) measurements of branches were found to be significantly affected by genotype, DAS and their interaction (p < 0.001, Table 2).

The genotypes with the greatest mean geodesic branch lengths at the conclusion of the experiment (35 DAS) were ‘ILL7537’ (532px), ‘PBA Hallmark XT’ (477px) and ‘Cumra’ (476px) whilst the lines with the smallest mean geodesic branch length were ‘Commondo’ (274px), ‘Aldinga’ (304px) and ‘Cobber’ (313px). As would be expected, mean geodesic branch lengths exceeded Euclidean branch lengths across the experiment. The correlation between geodesic and Euclidean distances was very strong (*R*^2^ = 0.9790902).

#### Splay and angle difference

**Fig. 8:**
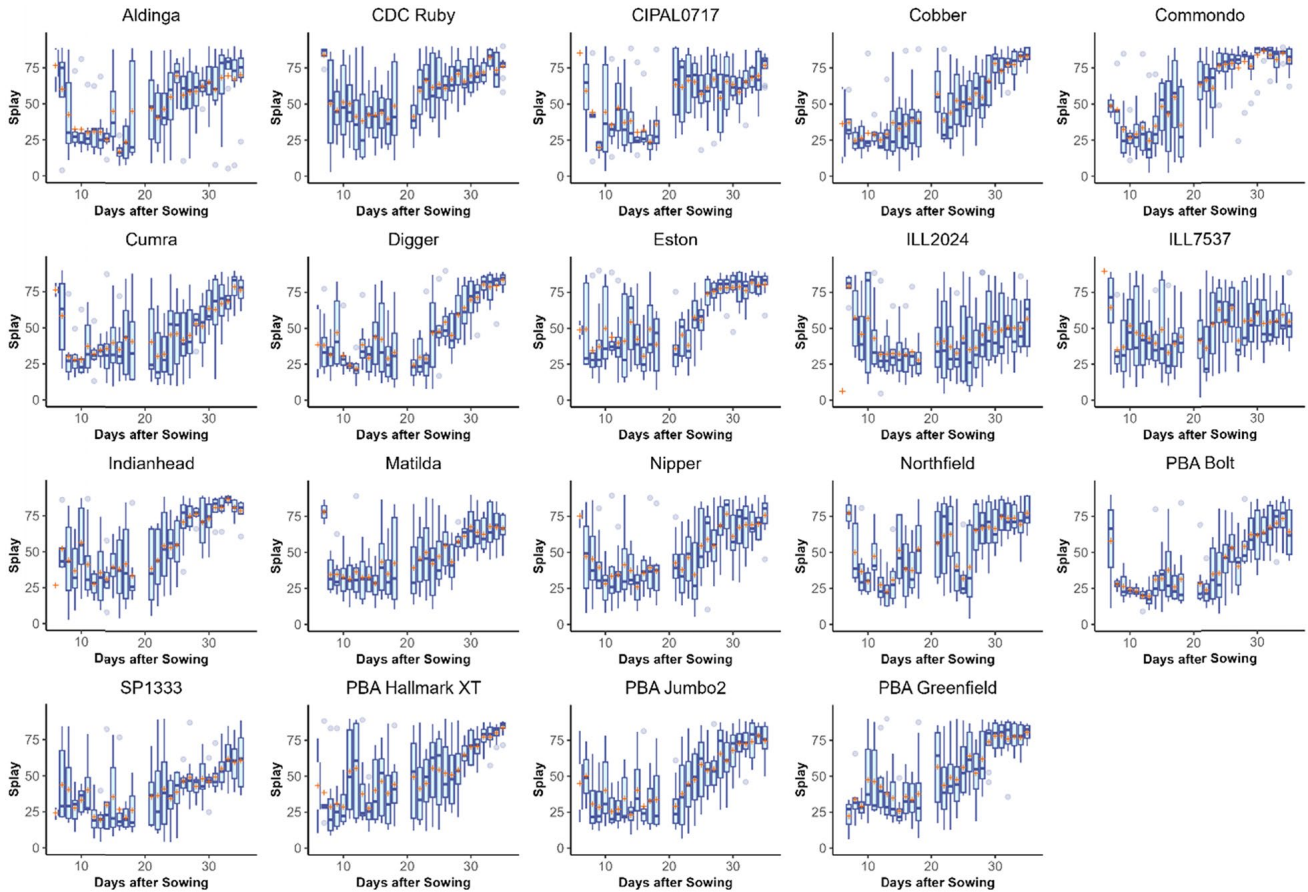
Box plots showing the distribution of branch splay counted by the branching algorithm in Experiment 1 for each lentil cultivar. The mean value is indicated with an orange cross. This figure shows the variability of branching architecture phenotypes between cultivars

**Fig. 9:**
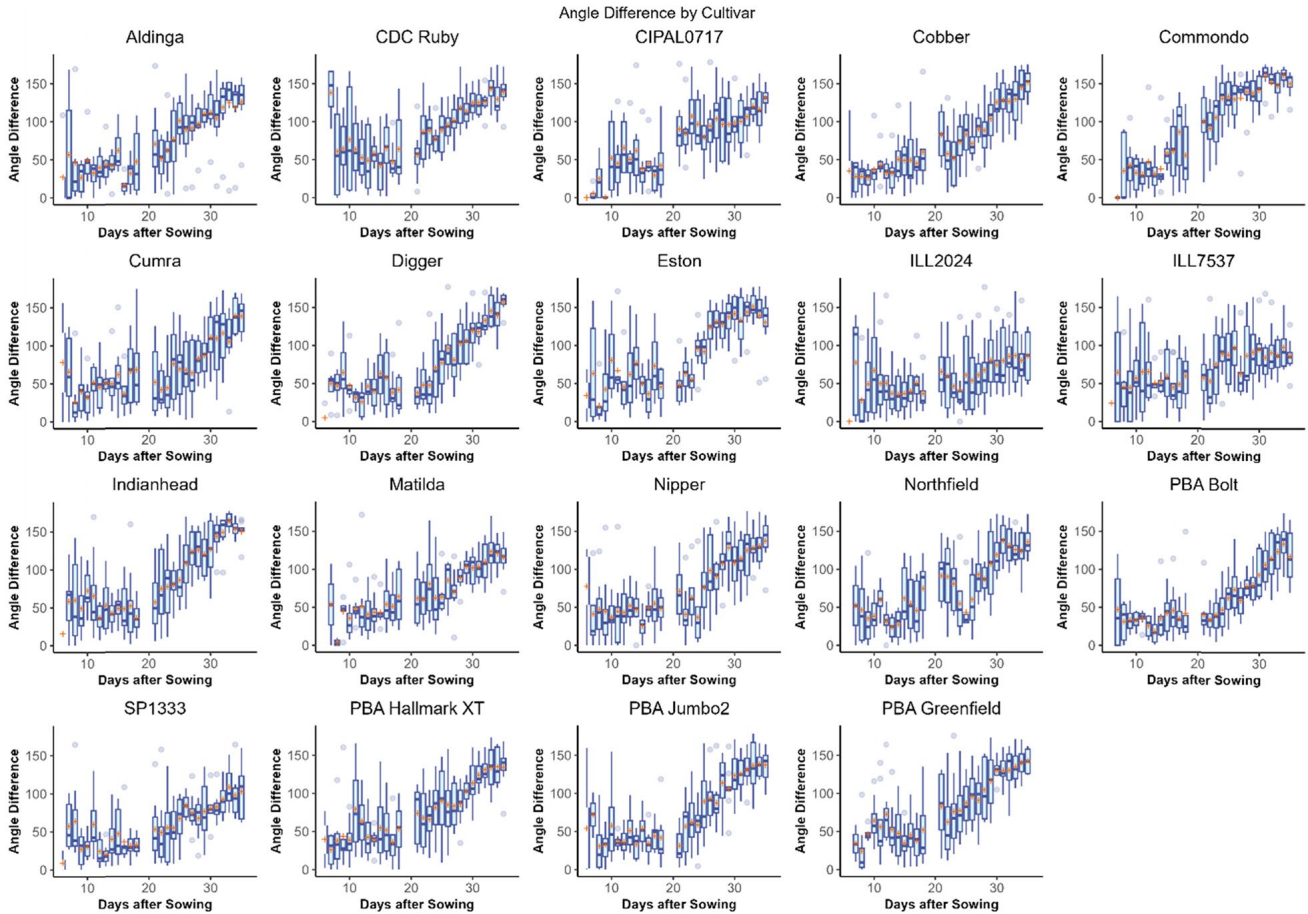
Box plots showing the distribution of angle difference counted by the lentil branching algorithm in Experiment 1 for each lentil cultivar. The mean value is indicated with an orange cross

The linear mixed-effects model in Eq. 1 was modified to predict splay and angle difference. The amount of variance explained by the model was 38% for splay and 50% for angle difference. These lower R^2^ values are likely due to the inherent difficulties in measuring these parameters. Within this model, the mean splay (Fig. 8) and angle difference (Fig. 9) measurements of branches were both found to be significantly affected by cultivar, DAS and their interaction (p < 0.001, Table 2).

The cultivars with the greatest mean splay at the conclusion of the experiment (35 DAS) were ‘PBA Hallmark XT’ (83.688°), ‘Digger’ (83.460°) and ‘Cobber’ (83.122°) whilst the smallest splay measurements were observed in ‘ILL7537’ (54.238°), ‘ILL2024’ (56.632°) and ‘SP1333’ (60.828°). In many cultivars, splay measurements were observed to be relatively stable until after Day 20 where they tended to increase as the lentil plants grew and developed (Fig. 8).

The greatest Angle Difference at 35 DAS was observed in ‘Digger’ (157.346°), ‘Cobber’ (151.649°) and ‘Commondo’ (149.900°) whilst the smallest Angle Difference was observed in ‘ILL7537’ (86.358°), ‘ILL2024’ (86.577°) and ‘SP1333’ (102.492°). The correlation between Splay and Angle Distance was strong (*R*^2^ = 0.8914198).

### Experiment 2

#### Number of branches

**Fig. 10:**
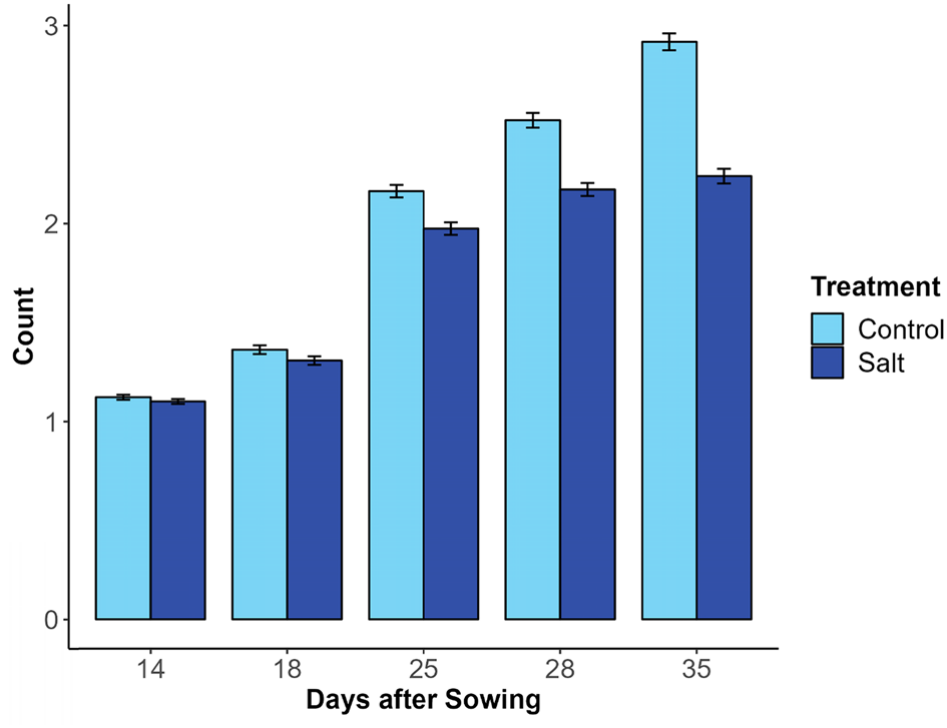
Bar chart showing the distribution of branch counts calculated by the lentil branching algorithm in Experiment 2 for each treatment group on days 14, 18, 25, 28 and 35. Error bars show 95% confidence interval

The branching algorithm was able to distinguish between salt-treated and untreated lentil lines based on differences in branch counts (Fig. 10). Between 14 and 35 DAS, the mean branch number was greater in the control treatments compared to the salt-treated plants (refer to SI04). At Day 35, the mean branch count for salt-treated plants was 2.24 compared to control plants with a mean branch count of 2.92.

**Fig. 11:**
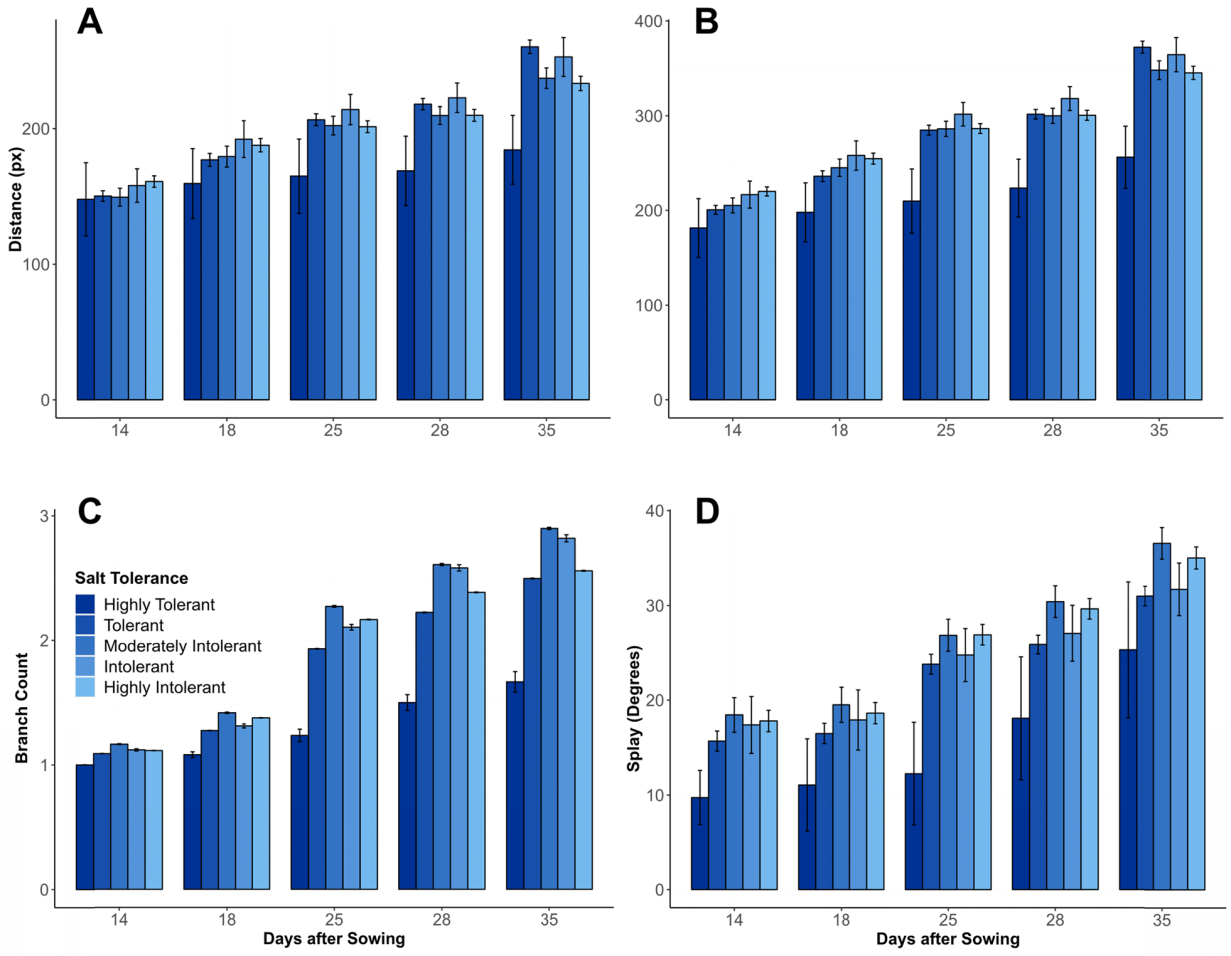
**A** Mean Euclidean branch length, **B** mean geodesic branch length, **C** mean branch number and **D** mean splay (**D**) calculated by the branching algorithm in Experiment 2 for plants grouped into classifications based on their reported salt tolerance as being “highly tolerant”, “tolerant”, “moderately intolerant”, “intolerant” or “highly intolerant” on days 14, 18, 25, 28 and 35. Error bars show the standard error to a 95% confidence interval

When the genotypes were classified into five groups based on their salt tolerance (as per the method described in [12], Fig. 11C, based on the “salt tolerance” data in SI02), DAS and salt tolerance classification were highly significant factors (p < 0.001). A pairwise t-test comparison of salt tolerance classifications at the conclusion of the experiment found no significant difference between the Moderately-Intolerant and Intolerant groups (p = 0.560, see Fig. 11C), and Highly-Intolerant and Tolerant groups (p = 0.057) but all other comparisons between the salt tolerance classification groups were significant.

#### Branch lengths

The amount of variance explained by the mixed-effects model was 57.8% for geodesic branch lengths and 46.5% for Euclidean branch lengths. Within this model, the mean geodesic and Euclidean distance (refer to SI04) measurements of branches were both found to be significantly affected by cultivar, DAS and treatment (p < 0.001, Table 3).

**Table 3:** Summary of analysis of variance (ANOVA, type II Wald chisquare test) results for Experiment 2

Response variable	R^2^	Factor	χ^2^	DF	Pr(> χ^2^)	*q*-value
Branch count	0.479	Cultivar	261.11	275	< 2.2e−16	3.3e−15
		DAS	29,914.92	1	< 2.2e−16	3.3e−15
		Treatment	513.06	1	< 2.2e−16	3.3e−15
Geodesic length	0.578	Cultivar	1144.5	275	< 2.2e−16	3.3e−15
		DAS	22,952.6	1	< 2.2e−16	3.3e−15
		Treatment	645.1	1	< 2.2e−16	3.3e−15
Euclidean length	0.465	Cultivar	1176.29	275	< 2.2e−16	3.3e−15
		DAS	12,430.78	1	< 2.2e−16	3.3e−15
		Treatment	579.74	1	< 2.2e−16	3.3e−15
Angle difference	0.530	Cultivar	844.721	275	< 2.2e−16	3.3e−15
		DAS	3599.953	1	< 2.2e−16	3.3e−15
		Treatment	29.048	1	7.061e−08	1.4122e−07
Splay	0.498	Cultivar	846.223	275	< 2.2e−16	3.3e−15
		DAS	2771.252	1	< 2.2e−16	3.3e−15
		Treatment	13.217	1	0.0002773	0.0002773

*q*-values are Holm [38] multiple test-corrected* p*-values

At Day 35, mean branch Euclidean distance was 271 px for control plants and 212 px for salt-treated plants, whilst mean geodesic length of branches was 395 px for Control plants and 307 px for salt-treated plants. As would be expected, mean geodesic branch lengths exceeded Euclidean branch lengths across the experiment. The correlation between geodesic and Euclidean distances was very strong (*R*^2^ = 0.9963578).

When the genotypes were classified into five groups based on their salt tolerance (as per the method described in [12], Fig. 11B, based on the “salt tolerance” data in SI02), DAS (p < 0.001) and salt tolerance classification (p = 0.00118) were highly significant factors. Plants classified as “highly tolerant” to salt exhibited reduced branching length in comparison to other classifications.

#### Splay

The amount of variance explained by the model was 38%. The lower R^2^ value is likely due to the inherent difficulties in measuring this parameter. Within this model, the mean splay (Fig. 11D) was found to be significantly affected by cultivar, DAS and treatment (p < 0.001, Table 3). Splay increased throughout the experiment for both treatments, but was less in salt-treated plants in the later part of the experiment. At Day 35, mean Splay was 34.0° for control plants and 32.6° for salt-treated plants.

When the genotypes were classified into five groups based on their salt tolerance (as per the method described in [12], Fig. 11D, based on the “salt tolerance” data in SI02), DAS (p < 0.001) and salt tolerance classification (p < 0.001) were highly significant factors. Plants classified as “highly intolerant” to salt exhibited reduced mean branch angle compared to other classifications.

## Discussion

This paper describes an algorithm that uses RGB imagery to reliably count the number of branches and measure their length and angle on glasshouse-grown lentil plants. This work is of importance because these are key traits-of-interest for lentil breeders owing to the potential of these traits to influence yield and harvestability [18, 19]. In recent decades, breeding efforts have resulted in the release of lentil cultivars with increased height and a reduced number of branches [3] which aids in mechanical harvesting and can lead to improved yield whilst reducing lodging [20]. However, the ability to screen diverse lentil lines and accurately phenotype their branch structures in a high-throughput quantitative manner has previously posed challenges, limiting the breeding of new cultivars that combine favourable architectural properties with other desirable traits to enhance productivity.

Our study presents results from two experiments. In the first, a diverse panel of 19 lentil genotypes were screened for innate differences in architectural traits. Our algorithm was able to discriminate between genotypes based on differences in the numbers of branches as well as their angle and length. In the second experiment, results comparing the effect of a salt treatment upon 190 diverse lentil genotypes, as a source of abiotic stress with the potential to affect plant architecture, was also reported to demonstrate an additional practical application for this work. We demonstrated that in the early stages of growth, our algorithm was able to distinguish between salt-tolerant and salt-stressed genotypes based on significant differences in their architecture that arose from the treatments. Both experiments demonstrate the utility of our approach to plant breeders who can use early-stage branch architectural traits as part of a pre-breeding programme. For instance, a high-throughput phenotyping screen utilising a glasshouse-based automated phenotyping platform could be conducted to identify and shortlist genotypes with desirable architectural traits. This approach would lead to a substantial reduction in labour and time taken to get a result whilst improving the quality of phenotypic data collected.

One of the key challenges for isolating branch structures from lentil plants is occlusion caused by overlapping branches and leaves. This is a well-documented problem with glasshouse- and field-based phenotyping systems, utilising 2D or 3D imagery [21, 22]. Our research utilised side-view images rather than overhead to better view branches, but as plants aged and their architecture became more complex, accuracy declined, as would be expected given the plant density of lentil plants. This was particularly evident after 35 DAS. Nevertheless, the methodology presented here can still be used for trait estimation in early growth stages. A positive correlation between plant growth and estimated branch number has been demonstrated using our methodology. The use of image-based trait estimation has assisted with crop improvement in other species such as wheat [23] and maize [24] and could be validly employed here too, for example, in estimating early vigour based on positive correlations in early growth stages.

Until now, there have been no reports of researchers extracting branching data from RGB images of plants grown on automated phenotyping platforms. Similar work has been focussed on unbranched monocotyledonous species such as barley, where 2D images were used to develop a model of leaf architecture [7] and maize [25, 26]. In dicotyledonous species, progress has been limited to the generation of 3D models of small seedlings in species such as *Arabidopsis* [27] and tomato [28].

The approach discussed in this research makes use of the basic functionality of a LemnaTec Scanalyser high-throughput automated phenomics platform and image analysis techniques utilising open-source software [13] in a Python coding environment, although the logic could be applied to other languages and applications. The methodology presented here is accessible to researchers because it avoids proprietary software and systems.

In terms of image analysis techniques, our research demonstrates an effective use of skeletonisation that is applied to visible-spectrum images generated by an automated glasshouse phenotyping platform. Whilst the use of skeletons to capture morphological characteristics of plants is widespread [29], this is more common with data generated from LiDAR laser scanners. For instance, skeletonisation has been successfully combined with 3D point clouds to analyse silique morphology in oilseed rape [30], map the architecture of fruit trees [31], identify the structure of individual corn plants in a field [32] and phenotype forest trees [33]. However, the application of skeletonisation as part of a morphological analysis pipeline using RGB images or images derived from visible-spectrum imagery is less common. Examples where this has been used include the phenotyping of roots [34], mapping of branching in edamame [35], the identification of legumes based on leaf venation [30], identification of the structure of maize tassels [36] and branches in roses for the purposes of training a robot to undertake pruning [37]. Our work is the first example of using skeletonisation from RGB images to map branching in dicotyledonous species. It is anticipated that the use of our algorithm could be readily expanded to other dicotyledonous species.

## Conclusion

Our approach demonstrates the use of a queue- and distance-based approach to identifying and quantifying branching structures in glasshouse-grown lentil plants using a high-throughput phenomics strategy that utilises open-source software to interpret skeletonised binary images. The algorithm measures the number of branches, their Euclidean and geodesic lengths and their angle using RGB images. The ability to collect such valuable phenotypic data from lentil plants could be used to guide future breeding work to improve traits such as lodging and yield.

### Supplementary Information

Supplementary Material 1: SI01. CSV file containing a list of the lentil cultivars used in Experiment 1 along with some phenotypic data.

Supplementary Material 2: SI02. CSV file containing a list of the lentil cultivars used in Experiment 2.

Supplementary Material 3: SI03. Box plots showing the distribution of Euclidean lengths of branches counted by the branching Algorithm in Experiment 1 for each lentil cultivar. The mean value is indicated with an orange cross.

Supplementary Material 4: SI04. Summary data for Experiment 2 containing measured data for branch number, mean geodesic branch length, mean Euclidean branch length and mean splay.

Supplementary Material 5: SI05. CSV file containing the Experiment 1 data.

Supplementary Material 6: SI06. CSV file containing the Experiment 2 data.

## Data Availability

The datasets supporting the conclusions of this article are included within this article’s additional files as SI05 (Experiment 1) and SI06 (Experiment 2).

## Abbreviations

CSV: Comma-separated value
DAS: Days after sowing
FIFO: First-in-first-out
JPEG: Joint Photographic Experts Group
PNG: Portable Network Graphic
RGB: Red–Green–Blue
RFID: Radio frequency identification
SE: Standard error of the mean
TV: Top view
uint16: Unsigned 16-bit
