# Supplementary figures and images for: Mapping and quantifying unique branching structures in lentil (Lens culinaris Medik.)
Source: Plant Methods. 2024 Jun 19;20:95. doi: 10.1186/s13007-024-01223-1 (PMC11188192; doi:10.1186/s13007-024-01223-1)

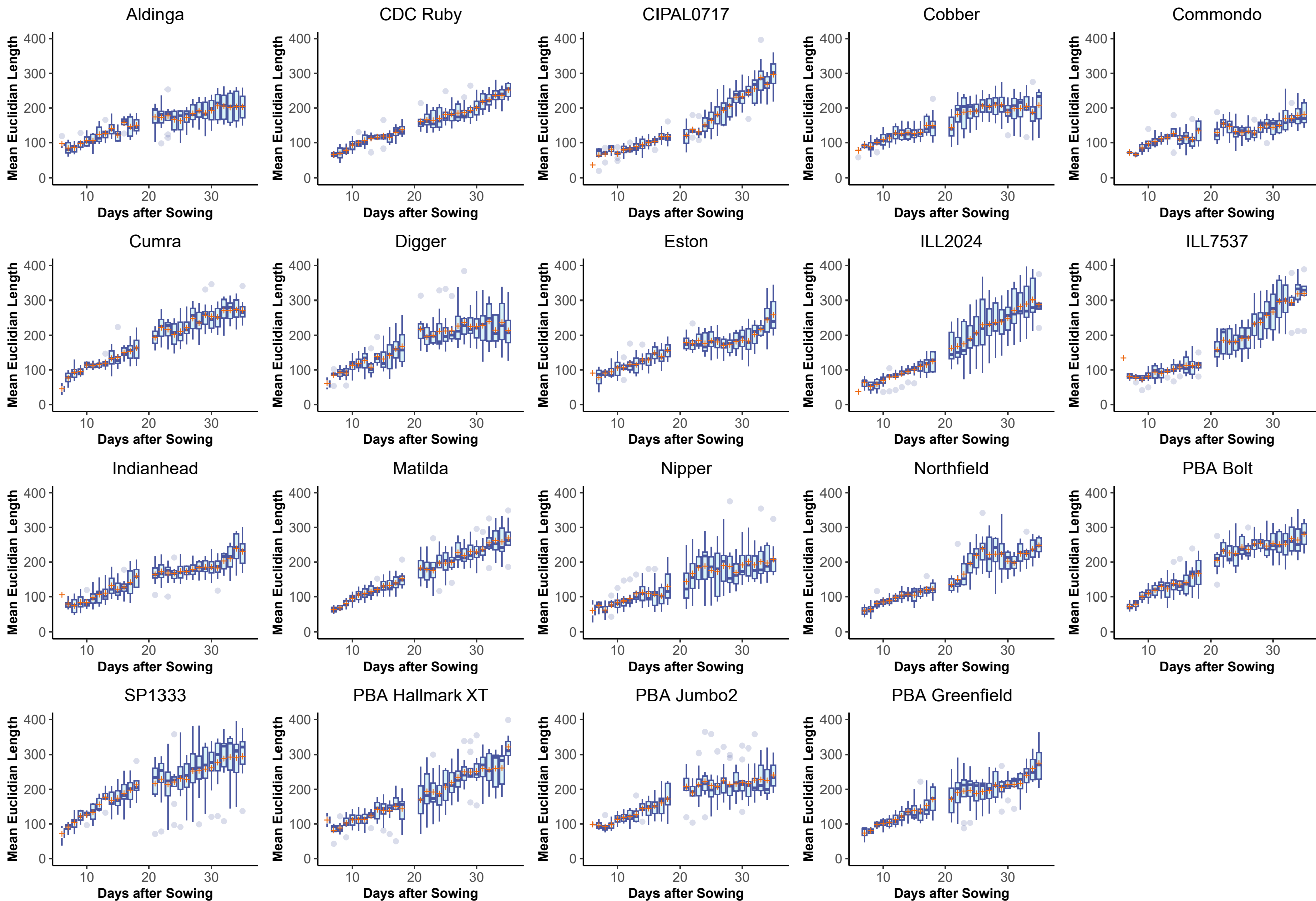

Supplement: Supplementary file 3 — Supplementary Material 3: SI03. Box plots showing the distribution of Euclidean lengths of branches counted by the branching Algorithm in Experiment 1 for each lentil cultivar. The mean value is indicated with an orange cross. [file 13007_2024_1223_MOESM3_ESM.pdf]
