# Supplementary material for: Mapping and quantifying unique branching structures in lentil (Lens culinaris Medik.)
Source: Plant Methods. 2024 Jun 19;20:95. doi: 10.1186/s13007-024-01223-1 (PMC11188192; doi:10.1186/s13007-024-01223-1)
Supplement: Supplementary file 4 — Supplementary Material 4: SI04. Summary data for Experiment 2 containing measured data for branch number, mean geodesic branch length, mean Euclidean branch length and mean splay. [file 13007_2024_1223_MOESM4_ESM.docx]

SI04 Summary data for Experiment 2 containing measured data for branch number, mean geodesic branch length, mean Euclidean branch length and mean splay.

| Days | Control | | | | | 100 mmol NaCl | | | | |
| --- | --- | --- | --- | --- | --- | --- | --- | --- | --- | --- |
|  | *n* | Mean Branches | Mean Geodesic Length | Mean Euclidean Length | Mean Splay | *n* | Mean Branches | Mean Geodesic Length | Mean Euclidean Length | Mean Splay |
| 14 | 906 | 1.12±0.01 | 210±4.5 | 155±3.9 | 17.5±1.1 | 900 | 1.10±0.01 | 209±4.3 | 155±3.6 | 16.5+1.0 |
| 18 | 904 | 1.36±0.02 | 245±6.0 | 181±5.1 | 18.3±1.1 | 902 | 1.31±0.02 | 246±5.8 | 183±4.9 | 17.4±1.1 |
| 25 | 902 | 2.16±0.03 | 295±6.8 | 212±5.7 | 24.7±1.3 | 901 | 1.97±0.03 | 276±6.3 | 195±5.3 | 26.3±1.4 |
| 28 | 908 | 2.52±0.04 | 318±7.2 | 226±5.9 | 27.8±1.4 | 905 | 2.17±0.03 | 282±6.5 | 199±5.5 | 28.3±1.5 |
| 35 | 910 | 2.92+0.04 | 395±9.7 | 271±7.5 | 34.0±1.5 | 904 | 2.24±0.04 | 307±7.9 | 212±6.3 | 32.6±1.6 |
